# Aneuvis: web-based exploration of numerical chromosomal variation in single cells

**DOI:** 10.1186/s12859-019-2842-1

**Published:** 2019-06-17

**Authors:** Daniel G. Piqué, Grasiella A. Andriani, Elaine Maggi, Samuel E. Zimmerman, John M. Greally, Cristina Montagna, Jessica C. Mar

**Affiliations:** 1 Department of Systems Biology, Albert Einstein College of Medicine, Bronx, NY 10461 USA; 2 Department of Genetics, Albert Einstein College of Medicine, Bronx, NY 10461 USA; 3 Department of Pathology, Albert Einstein College of Medicine, Bronx, NY 10461 USA; 4 Department of Epidemiology and Population Health, Albert Einstein College of Medicine, Bronx, NY 10461 USA; 5 Australian Institute for Bioengineering and Nanotechnology, the University of Queensland, Brisbane, QLD 4072 Australia

**Keywords:** Aneuploidy, Single-cell genomics, Numerical chromosomal variation, Fluorescence in situ hybridization, Visualization, Automated hypothesis testing, Multi-experiment comparison

## Abstract

**Background:**

Numerical chromosomal variation is a hallmark of populations of malignant cells. Identifying the factors that promote numerical chromosomal variation is important for understanding mechanisms of carcinogenesis. However, the ability to quantify and visualize differences in chromosome number between experimentally-defined groups (e.g. control vs treated) obtained from single-cell experiments is currently limited by the lack of user-friendly software.

**Results:**

Aneuvis is a web application that allows users to determine whether numerical chromosomal variation exists between experimental treatment groups. The web interface allows users to upload molecular cytogenetic or processed single cell whole-genome sequencing data in a cell-by-chromosome matrix format and automatically generates visualizations and summary statistics that reflect the degree of numeric chromosomal variability.

**Conclusions:**

Aneuvis is the first user-friendly web application to help researchers identify the genetic and environmental perturbations that promote numerical chromosomal variation. Aneuvis is freely available as a web application at https://dpique.shinyapps.io/aneuvis/ and the source code for the application is available at https://github.com/dpique/aneuvis.

**Electronic supplementary material:**

The online version of this article (10.1186/s12859-019-2842-1) contains supplementary material, which is available to authorized users.

## Background

Alterations in chromosome number are a hallmark of cancer [1]. Within a population of single cells, increased numerical chromosomal variation may reflect underlying whole chromosomal instability (W-CIN) [2], which promotes chemotherapy resistance [3]. The process of identifying numerical chromosomal variation in single cells is thus important, among other reasons, for understanding how cancers become resistant to chemotherapy. This process can be divided into two steps. The first step is to quantify the number of chromosomes per cell. Multiple experimental techniques and computational tools exist for completing this step, and the final output is often a spreadsheet or text file that contains chromosomal copy number information for all nuclei analyzed [4]. The second step is to quantify the degree of numerical chromosomal variation. Existing approaches, such as AneuFinder, estimate the degree of numerical aneuploidy from single cell whole genome sequencing (sc-WGS) data but require knowledge of the R programming language [5]. In addition, existing approaches do not allow specification and comparison of experimental treatment groups. Furthermore, no freely-available software exists for the processing of chromosomal count data derived from locus specific fluorescent in situ hybridization (FISH) or spectral karyotyping (SKY).

Here, we introduce aneuvis, a user-friendly web application for visualizing and summarizing numerical chromosomal variability in populations of single cells belonging to experimentally-defined treatment groups. Aneuvis operates downstream of existing experimental and computational approaches that generate a matrix containing the estimated chromosomal copy number per cell. Users upload both a copy number matrix along with a key that links individual cells with experimental groups. Aneuvis is the first freely-available, user-friendly application to automatically calculate metrics and generate graphics that reflect numerical chromosomal variation between experimental treatment groups. Aneuvis is available to be used as a stand-alone web application [6].

## Results

### Aneuvis design and workflow

**Fig. 1:**
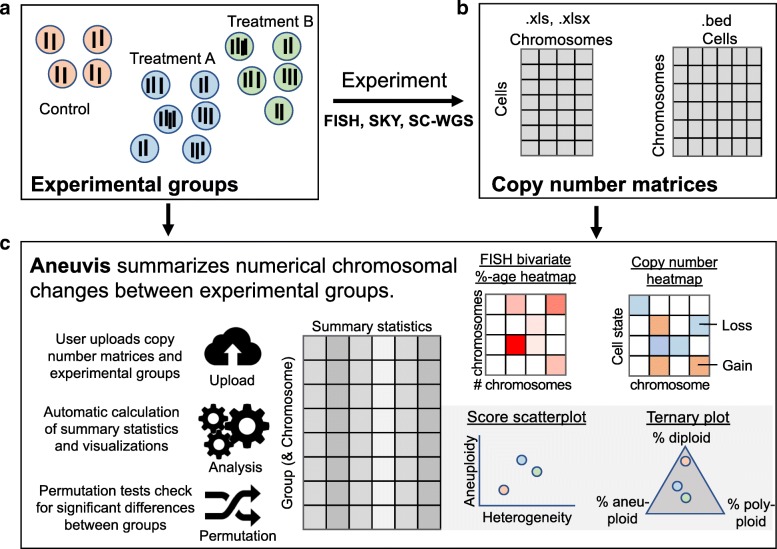
Overview of the aneuvis workflow for analyzing numerical chromosomal variation. **a** An experiment begins with the quantification of number of chromosomes per cell using either FISH, SKY, or sc-WGS. **b** Next, the number of chromosomes per cell within each treatment group is stored as a cell x chromosome matrix, where the entries indicate the number of inferred copies of a chromosome in a cell. **c** Aneuvis incorporates information from the experimental design as well as from chromosomal copy number matrices to determine whether differences exist between treatment groups. A table of descriptive statistics summarized by group and by chromosome is automatically generated and available for download. Visual representations of the relationship in aneuploidy between different groups are also automatically generated. A permutation-based approach allows the user to conclude whether there is a statistically significant difference in the ploidy characteristics between treatment groups

**Fig. 2:**
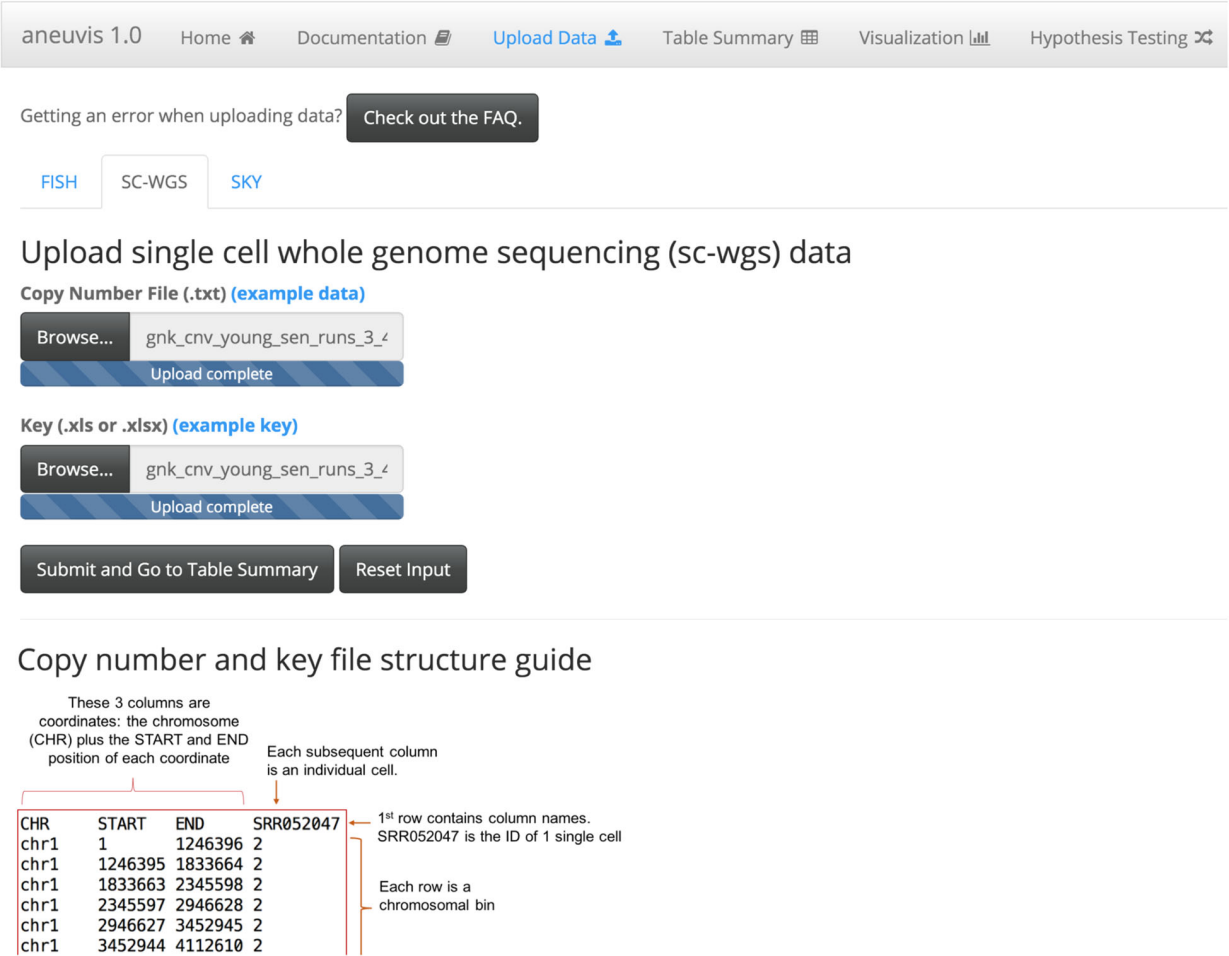
Screenshot of the aneuvis data upload interface. The top half of the screen is where the user uploads the data into aneuvis. The bottom half of the interface contains a guide that describes how each file should be formatted for input into aneuvis (here, a guide for formatting sc-WGS data processed by Ginkgo)

**Fig. 3:**
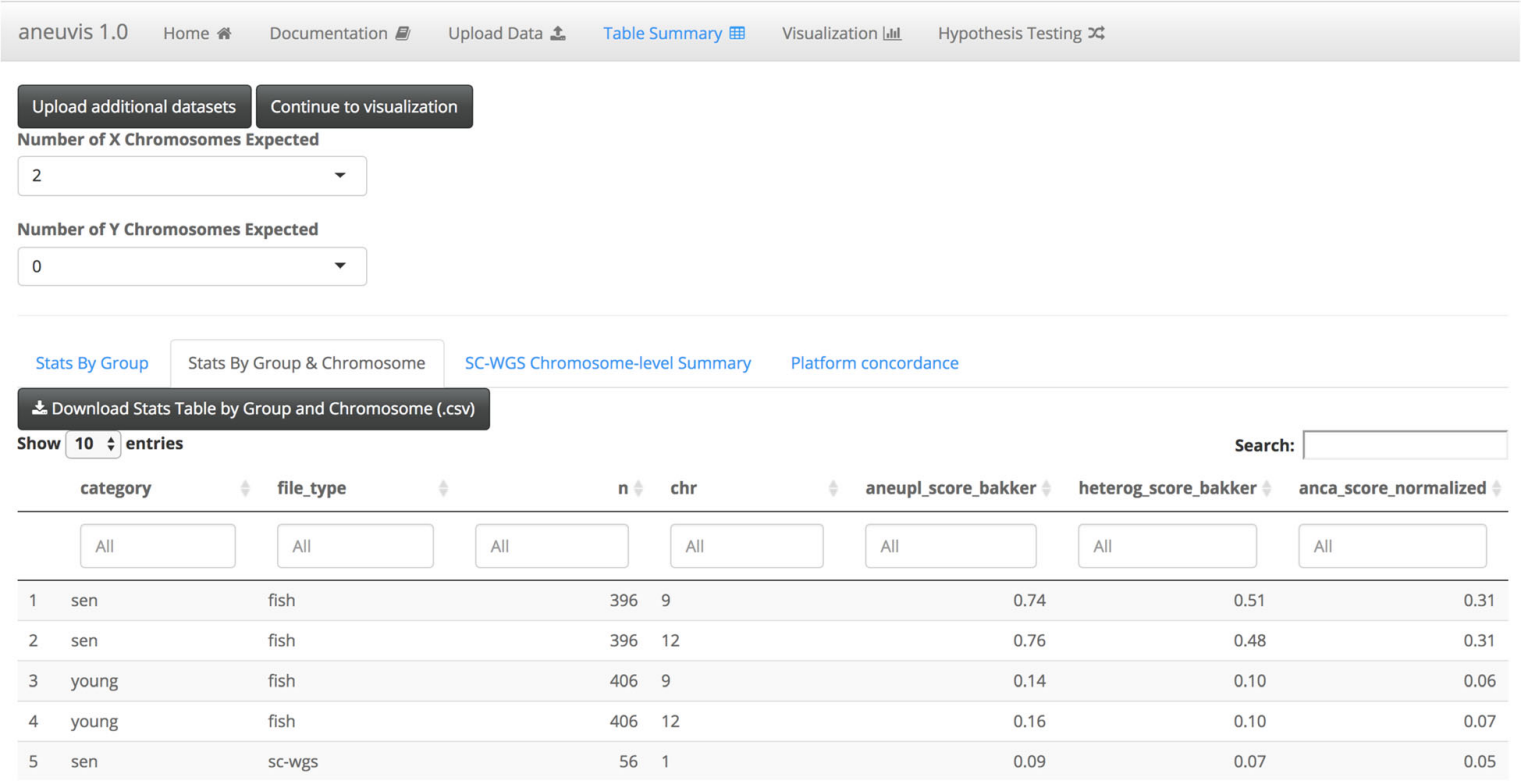
Screenshot of table summary available in aneuvis. Each row displays summary statistics for quantifying numerical chromosome variation per chromosome, treatment, and experimental type. Tables are interactive and can be filtered, sorted, or downloaded

Aneuvis facilitates the analysis of numerical chromosomal variation between experimental treatment groups and works downstream of existing approaches that quantify copy number changes in single cells (Fig. 1). The aneuvis workflow begins with the upload of one of three types of data (sc-WGS, FISH, or SKY) via a graphical user interface (Fig. 2). File formatting guidelines are provided for each data type (see Fig. 2 for an example of the specifications for processed single cell WGS data). Upon clicking the submit button, aneuvis automatically generates output that is divided into three sections – table summary, visualization, and hypothesis testing – that are listed along the navigation bar within aneuvis (Fig. 2, top section). The user is first taken to the “Table Summary” section. The purpose of the table summary section is to quantify the degree of chromosomal variability per experimental group using six different literature-derived statistics (see Table 1 for a description of the statistics). The table summary is divided into two parts – aggregate and chromosome-level summaries per group. The same statistics (except for the ploidy proportion and instability index, which are features of population of cells) are calculated at the level of the treatment group (aggregate) as well as at the chromosomal level to identify chromosomes that are the most perturbed within and between each group. The user interface to the summary table is dynamic and searchable, and the data is downloadable. Figure 3 shows a screenshot of the table summary output.

**Table 1:** Scores and indices used to quantify aneuploidy

Method	Equation	Support	%-based?	Reflects magnitude of copy number changes?	Reflects variability in chromosomal states?	Meaning and Considerations
Instability index (***I***)	$I=\left(\frac{\sum \limits_{t=1}^T{k}_t}{T}\right)\ast 100$	*I* ∈ [0, ∞)	No	No	Yes	Calculates the sum of the percentage of abnormal chromosome counts across each chromosome.
						Consider: *I* may increase with the # of chromosomes measured.
ANCA (***A***)	$A=\left(\frac{\sum \limits_{n=1}^N{q}_n}{n}\right)$	*A* ∈ [0, ∞)	No	No	No	ANCA = Average Number of Copy Number Alterations per cell.
						Consider: *A* will ↑ as the # of measured chromosomes ↑s.
Normalized ANCA (***AN***)	$AN=\left(\frac{\sum \limits_{n=1}^N{q}_n}{T\ast n}\right)$	*A* ∈ [0, ∞)	No	No	No	*AN* is the ANCA score normalized for differences in number of chromosomes measured per experimental type (eg FISH vs sc-WGS).
Aneuploidy score (***D***)	$D=\frac{1}{TN}\sum \limits_{n=1}^N\sum \limits_{t=1}^T\mid {c}_{n,t}-{e}_t\mid$	*D* ∈ [0, ∞)	No	Yes	No	*D* increases with increased chromosome number.
						Consider: Even a few cells with very large numbers of chromosomes will cause *D* to ↑ (not so with *H*)
Heterogeneity score (***H***)	$H=\frac{1}{TN}\sum \limits_{t=1}^T\sum \limits_{f=0}^{S-1}f\bullet {m}_{f,t}$	*H* ∈ [0, ∞)	No	No	Yes	Increases with greater ‘spread’ in the number of observed chromosomal states
						Consider: *H* is maximized when each cell has a different number of chromosomes, regardless of absolute # of chromosomes.
Ploidy proportion	*P*_*A*_ + *P*_*D*_ + *P*_*P*_ = 1	*P*_*A*, *D*, *P*_ ∈ [0, 1]	Yes	No	Yes	*P*_*D*_ = 1, *P*_*A*_ = 0, *P*_*P*_ = 0 when population is diploid.

For the instability index [7], ***T*** = the number of unique chromosomes examined. ${\boldsymbol{k}}_{\boldsymbol{t}}=\frac{\mathbf{1}-\hat{\boldsymbol{p}}_{\boldsymbol{t}}}{\boldsymbol{N}}$, where ***p*^**_***t***_ is the number of cells containing the modal number of the ***t***^***th***^ chromosome. ***N*** is the number of cells examined. For the ANCA score [8, 9], ***q***_***n***_ is the number of non-diploid chromosomes observed in the ***n***^***th***^ cell. For the aneuploidy score [5], ***c***_***n,t***_ is the copy number of the ***n***^***th***^ cell at the ***tt***^***th***^ chromosome. ***e***_***t***_ is the euploid copy number at the ***t***^***th***^ chromosome. For the heterogeneity score [5], ***S*** is the total number of copy number states. ***m***_***f,t***_ is the number of cells with copy number state ***s*** at bin ***t***. ***m***_***f,t***_ is ordered such that ***m***_***f =*** **0*,t***_ ***≥ m***_***f =*** **1*,t***_ ***≥***  …  ***≥ m***_***f = S −*** **1*,t***_. For the ploidy proportion, ***P***_***A***_ is the proportion of cells that are aneuploid, ***P***_***D***_ is the proportion of cells that are diploid, and ***P***_***P***_ is the proportion of cells that are polyploid

**Fig. 4:**
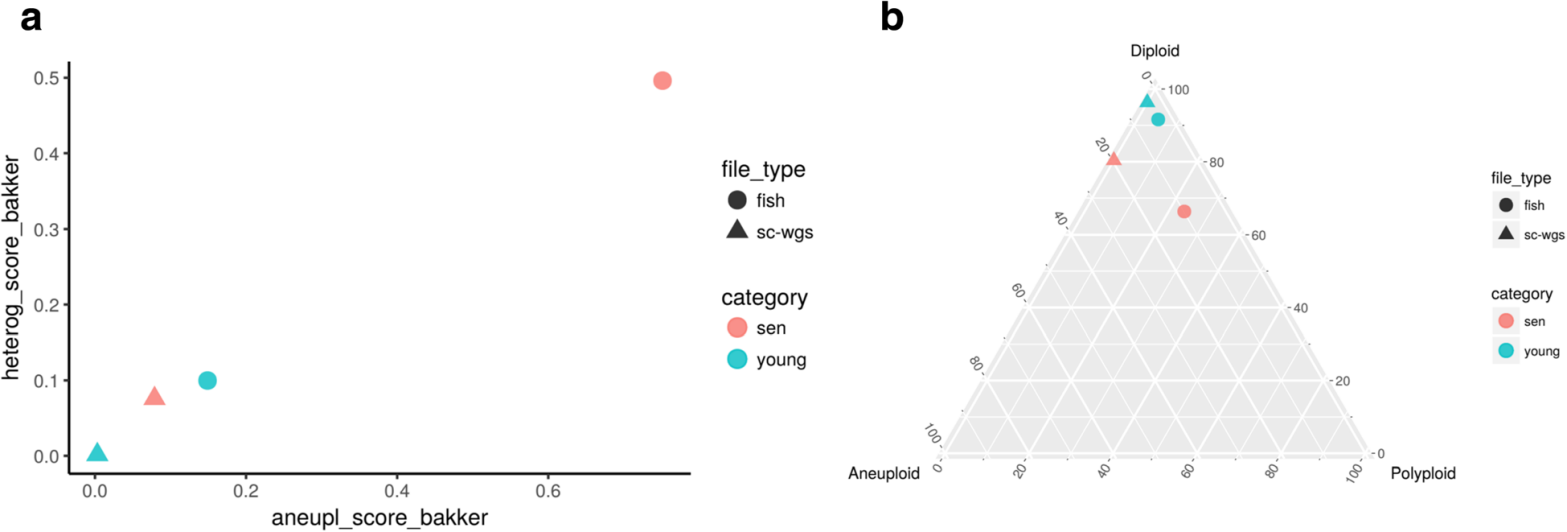
Screenshot of scatterplot and ternary plot using data generated from the table summary. **a** The relationship between the aneuploidy score (x-axis) and the heterogeneity score (y-axis) is shown for data derived from FISH (circles) and sc-WGS (triangles). Senescent fibroblasts are colored red and young cells are colored turquoise. **b** A ternary plot showing the percentage of cells that are diploid, polyploid, and aneuploid per experimental approach and per treatment category

**Fig. 5:**
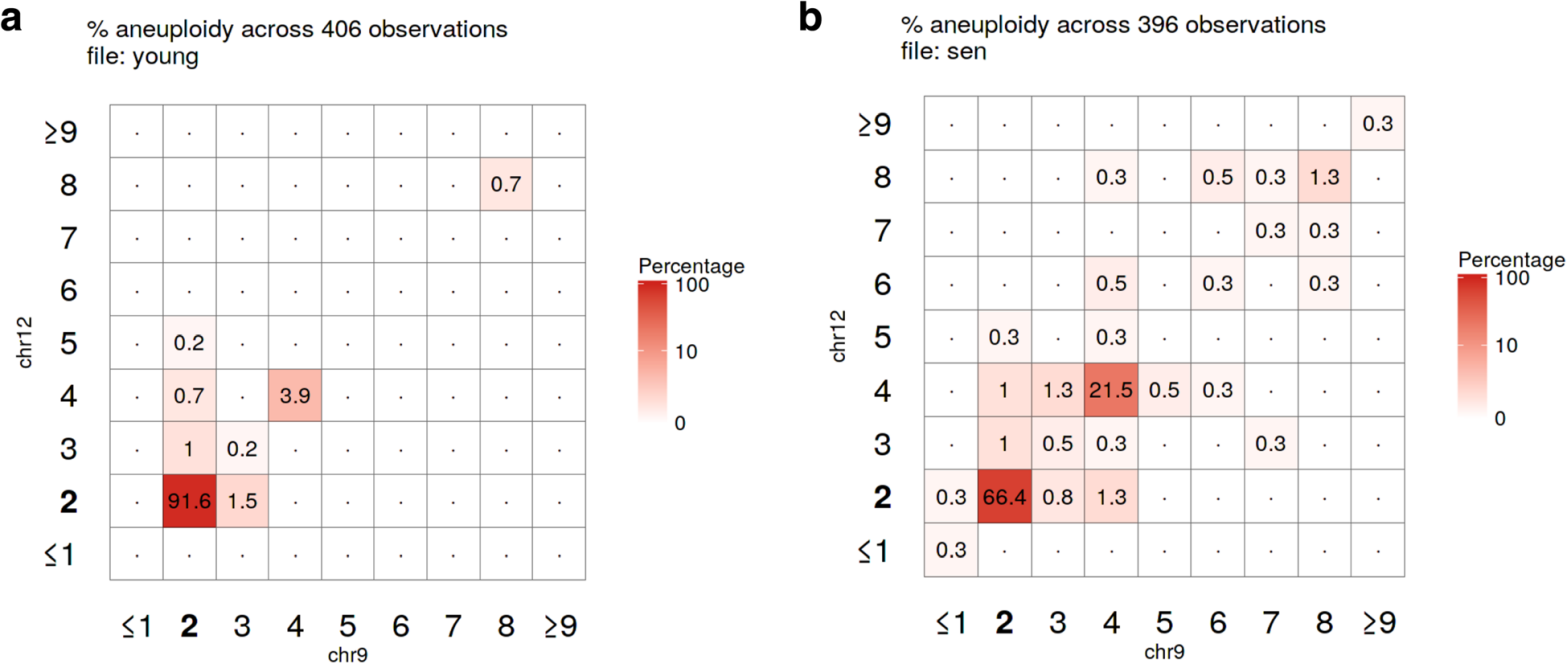
Screenshot of the aneuvis bivariate percentage heatmaps from 2-chromosome FISH data. The percentage of (**a**) young (*N* = 406) or (**b**) senescent (*N* = 396) fibroblasts within each chromosomal state is shown. The depth of the red color indicates that more cells exist within the indicated state. The axes for the diploid state (2 copies of each chromosome) are highlighted in bold

Using the statistics derived from the table summary, aneuvis automatically generates visualizations that compare experimental groups and that are data type-specific within the “Visualization” section (see Fig. 1 for overview). Data visualizations (e.g. scatterplots) shared across all experimental inputs are further divided into group and chromosomal level summaries. Group level summaries include an interactive scatterplot showing the bivariate relationship between the degree of chromosomal variability (heterogeneity score) and the severity of numerical aneuploidy (aneuploidy score) (Fig. 4a). Furthermore, ternary plots [10] show the proportion of cells that are diploid, polyploid, and aneuploid (Fig. 4b). Both plots include experimental groups from all uploaded data types, thus enabling comparisons between experimental inputs, such as between FISH and single cell whole genome sequencing (sc-WGS). The chromosome level summary includes an interactive scatterplot showing the relationship between heterogeneity and aneuploidy scores. The visualizations that are data type-specific include summarized copy number heatmaps for SKY and sc-WGS data and a novel bivariate percentage heatmap for FISH data (Fig. 5). To our knowledge, the bivariate percentage heatmap is the first such published visualization of chromosomal ploidy from FISH data in a population of cells. Finally, the visualizations can be downloaded as a pdf file from aneuvis using the “Download PDF” button, and an example is shown in Additional file 1.

### Hypothesis testing

Oftentimes, a research question may involve asking whether two or more treatment groups are different from each other in terms of their degree of chromosomal variability. Permutation testing allows researchers to test statistical hypotheses using an intuitive, distribution-free approach. Within the application, users can test the null hypothesis that each group has the same value of the test statistic relative to all other groups. The user selects the number of permutations and the test statistic to be permuted. A threshold for rejecting the null hypothesis is set at a *P*-value (or Benjamini-Hochberg adjusted q-value [11]) of 0.05. Aneuvis generates a summary table of permutation test statistics, as well as a heatmap representation of pairwise relationship between different treatments. To our knowledge, this is the first web-based platform that allows researchers to perform null hypothesis significance testing between experimental treatment groups based on differences in chromosomal copy number.

### Usage scenario

Senescence of mammalian cells is associated with numerical chromosomal instability in vitro, as assessed by four-color interphase FISH [12]. To demonstrate the utility of aneuvis, the copy number status of high-passage IMR90 fibroblasts (i.e. senescent fibroblasts) were compared with low-passage fibroblasts (i.e. young fibroblasts) using two techniques: four-color interphase FISH and sc-WGS (see Methods). Automated hypothesis testing using a permutation-based approach within aneuvis revealed that senescent fibroblasts were significantly different from young fibroblasts in terms of the aneuploidy and heterogeneity scores [5] derived from FISH (500 permutations, *P*-value = 0.002) but not from sc-WGS (500 permutations, P-value > 0.05). These results were supported by the FISH bivariate heatmaps, which show increased numerical chromosomal variation in senescent fibroblasts relative to young fibroblasts (Fig. 5). Inconsistencies between FISH and sc-WGS in measuring aneuploidy are recognized, and likely due to a differential sensitivity of these techniques (FISH is prone to the detection of false positives and sc-WGS is prone to false negative CNV detection) [4]. However, the observed aneuploidy and heterogeneity scores were higher in senescent versus young fibroblasts for both FISH and sc-WGS inputs (Fig. 4), highlighting a trend toward increased numerical chromosomal variability in senescent fibroblasts that was present across both FISH and sc-WGS. Existing methods that involve a graphical user interface for visualizing single cell copy number data do not support this type of quantitative and comparative cross-platform analysis (Table 2). These results highlight the ability of aneuvis to quantitatively integrate results from multiple experimental platforms and to deliver a multidimensional perspective of numerical chromosomal variation in populations of senescent cells.

**Table 2:** Active applications for analyzing and visualizing copy number variation data with graphical user interfaces (GUI)

Application	Reference &/or URL	Application Type	Data type	QDV
CNVinspector	[13, 14]	Web application	Array CGH	No
Ginkgo	[15, 16]	Web application	sc- WGS	No
GenomeCAT	[17, 18]	Java application	Array CGH, WGS	No
SNPitty	[19, 20]	Docker container	WGS	No
Aneuvis	[6]	Web application	sc-WGS^a^, SKY, FISH	Yes

Each row contains data for a separate application, and columns specify features of each application. The URL for the application or source code, application type, and data type used are listed for each application. The capacity of the application to quantify the degree of numerical chromosomal variation (QDV) within and between treatment groups is also listed

^*a*^Requires preprocessing from aligned .bam or .bed files into a copy number state matrix by an application such as Ginkgo

### Comparison of aneuvis to the existing web-based method for sc-WGS analysis (Ginkgo)

**Fig. 6:**
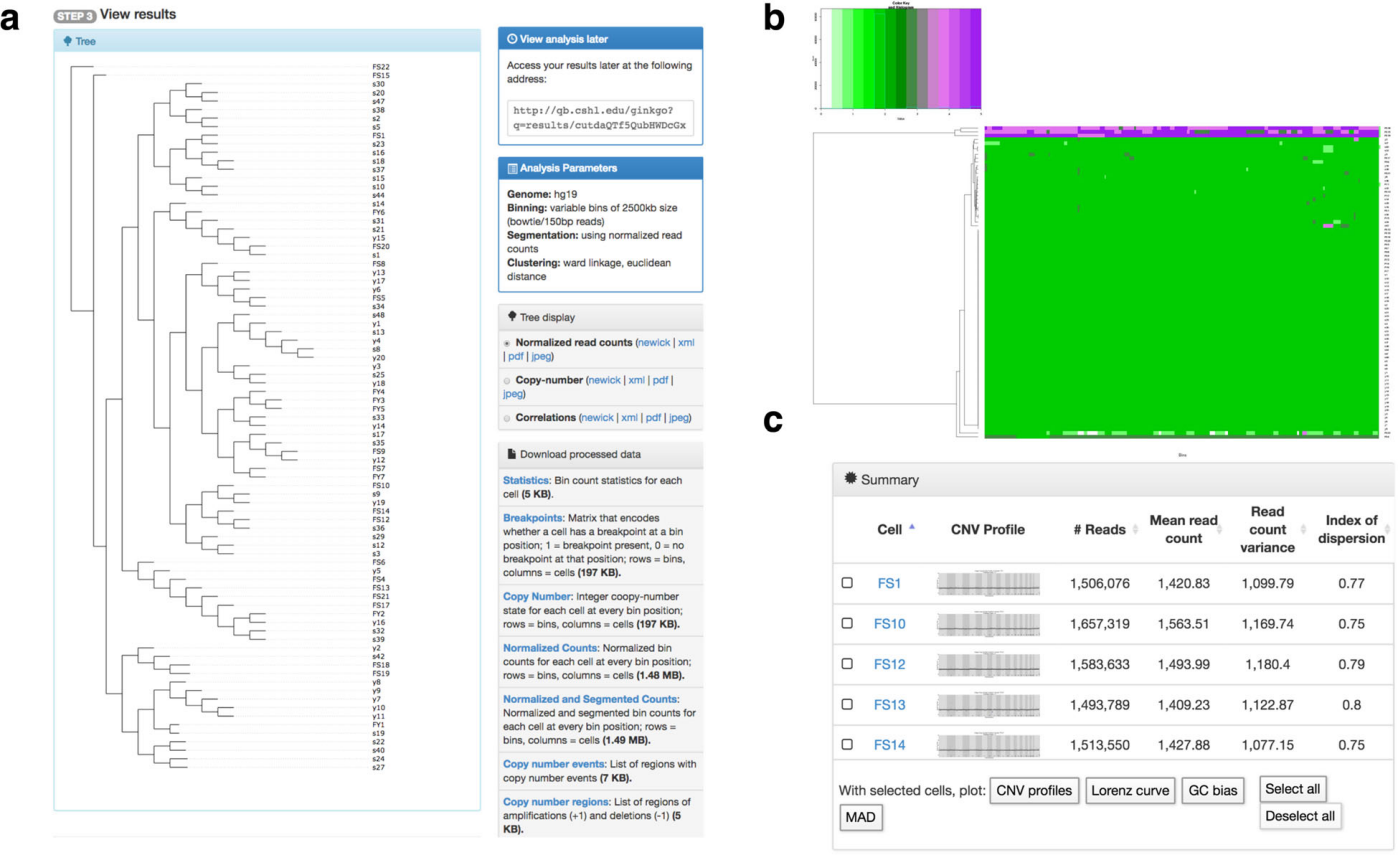
Screenshots of Ginkgo visualization and output from sc-WGS input from 83 single cells. **a** Dendrogram representation based on unsupervised hierarchical clustering using Ward linkage and the Euclidian distance of copy number states between individual cells. The lower right side of panel A contains links to download the summarized copy number data. **b** An example of a copy number heatmap constructed by aneuvis. Each row corresponds to an individual cell, and each column corresponds to a genomic bin. **c** Summary section showing various quality control summaries alongside graphical outputs of the copy number state across the genome of each cell

Next, we wanted to demonstrate how aneuvis compares against Ginkgo, the only other existing web-based method for analyzing single-cell WGS data [15]. To do this, the same set of sc-WGS data from 83 single cells was run through Ginkgo (see Methods). The main differences between the Ginkgo and aneuvis output are described below, and a more detailed description of Ginkgo outputs is available in the Methods section (also see Fig. 6a-c). Most notably, Ginkgo quantifies the copy number state in single cells from aligned sequencing reads, performs unsupervised analysis (e.g. clustering) of copy number state, and provides a series of visualizations showing similarities between cells based on the copy number state (e.g. copy number heatmap, dendrogram). However, Ginkgo does not perform comparative analyses between predefined experimental groups (e.g. young versus senescent fibroblasts). Aneuvis is designed to perform comparative analyses between predefined experimental groups and, in contrast to Ginkgo (which processes sc-WGS data only), aneuvis summarizes copy number variability from multiple types of data (e.g. sc-WGS, FISH, and SKY) (Fig. 1). Within the dendrograms and copy number heatmaps generated by Ginkgo, the copy number differences between the 27 young and 56 senescent fibroblasts were difficult to appreciate (Fig. 6a-b). Furthermore, Ginkgo does not provide users with statistical testing to determine whether experimental treatment groups have significantly different copy number profiles. In contrast, aneuvis provides statistical testing within the application using a rigorous permutation-based approach. As with Ginkgo, the quantitative summaries from aneuvis between treatment groups are available in a user-friendly format for download and include both graphical visualizations and data tables. In summary, aneuvis provides users with a set of unique visualizations and summary statistics for processed sc-WGS copy number matrices that complement the analyses in existing web-based applications. Aneuvis works together with Ginkgo’s copy number output, among other data types, to provide a user-friendly and comprehensive web interface for quantifying differences in chromosomal copy number between experimental groups.

## Discussion

Copy number changes in both somatic and germline cells are thought to be important drivers in cancer, aging, environmental health, and reproductive development [2]. For example, older mothers are more likely to have a child with a trisomy disorder (e.g. Down syndrome), and this is thought to be due to an increase in chromosomal segregation errors within aging oocytes [21]. Understanding the factors that lead to accelerated rates of chromosomal missegregation in individual cells could have important implications for human health and policy. For example, previous studies have found associations between environmental chemicals (e.g. pesticides) and increased rates of chromosomal missegregation [22]. To identify additional stressors, study designs that incorporate predefined exposure groups will be needed to identify whether an exposure alters chromosomal copy number. However, no user-friendly platforms exist to facilitate such a comparison. Aneuvis is the first web-based application to address the need for performing quantitative comparisons of chromosomal copy number variation between defined experimental groups.

Oftentimes, multiple experimental techniques are used for quantifying copy number changes in cells in response to a stressor. This may be necessary to demonstrate experimental rigor and to provide a complementary approach whereby the shortcomings of one method (e.g. low genome coverage of FISH) are addressed by the strengths of another (e.g. the whole genome coverage of sc-WGS). Tools with a user-friendly interface that integrate chromosome copy number information from multiple methods are lacking. In the present study, aneuvis both highlighted differences in the results obtained from different methods (FISH vs. sc-WGS) and demonstrated a cross-platform trend toward increased numerical chromosomal variation in senescent cells relative to non-senescent cells. Existing methods for copy number analysis (e.g. Ginkgo) are unable to integrate copy number information from multiple sources.

## Conclusions

Aneuvis is the first web-based application developed to automatically summarize numerical chromosomal variation in single cells between experimental treatment groups. We demonstrate the utility of aneuvis by analyzing the chromosomal copy number status of young and senescent fibroblasts obtained using two techniques: four-color interphase FISH and sc-WGS. The results from aneuvis show that the differences in W-CIN between treatment groups depend on the experimental method used, and that an integrated framework like aneuvis can highlight trends between complimentary experimental methods performed on the same experimental treatments. Aneuvis is also the first web-based tool to quantify and visualize numerical chromosomal variation from multiple data types. Aneuvis provides a comprehensive approach to visualizing and quantifying copy number variation between experimental treatment groups, the first time that such a tool has been made available. In summary, aneuvis is a user-friendly, web-based, and open-source tool that will enable researchers to identify novel mechanisms underlying the generation of numerical chromosomal variation.

## Methods

### Indices for measuring numerical chromosomal variation

The instability index (I) is a metric that calculates the percentage of cells that contain a chromosomal aberration [7]. This metric does not directly depend on the number of chromosomes; however, measuring more chromosomes may increase the likelihood of detecting at least one chromosome that contains an abnormal number of copies.

The Average Number of Copy Number Alterations (ANCA) score has been applied in the context of colorectal and cervical cancer in an attempt to quantify the relationship between tumor aggressiveness and genomic instability [8, 9]. Previous studies have uncovered that more aggressive tumors have a higher ANCA score. However, one limitation of the ANCA score is that it does not account for the number of chromosomes examined. Within aneuvis, we introduce a derivative of the ANCA score, called the Normalized ANCA score, which accounts for the number of chromosomes measured and enables comparisons of this metric between experiments that utilize different numbers of probes.

The aneuploidy (D) and heterogeneity (H) scores were derived from Bakker et al. and represent a pair of statistics that account for the number of cells and chromosomes tested for [5]. The aneuploidy score increases with an increased chromosome copy number – the only score to take the actual number of chromosomes into account. The heterogeneity score increases with the number of distinct chromosomal states observed, and is maximized when each cell has a distinct state. In contrast to the aneuploidy score, the heterogeneity score does not incorporate the chromosomal copy number. These statistics were derived for summarizing copy number data from whole genome single cell sequencing, though their flexible formulation enables them to be applied to other datasets.

In a cell, there are three possible states that a set of chromosomes can assume. Diploidy refers to the presence of two copies of each autosome in a cell, and is the physiologic state of most non-cancerous human cells. Polyploidy refers to an integer-valued increase in the number of chromosomes, often resulting from whole-genome duplication. Aneuploidy occurs when the copy number of 1 or more chromosomes differs from the others and is a feature of many cancers.

### Bivariate percentage heatmap

The bivariate percentage heatmap is used for visualizing the covariation between the counts of two chromosomes in a population of single cells. Each square within the grid represents the percentage of cells observed with a certain number of chromosomes listed on the X and Y axes. This approach is appropriate for FISH data, where the ploidy of cells is inferred from chromosome-specific fluorescent probes. For FISH data that include measurements from > 2 chromosomes, multiple bivariate plots are produced in aneuvis to account for all possible pairwise combinations of chromosomes. For example, a population of cells where 4 chromosomes were measured would generate $\left(\genfrac{}{}{0pt}{}{4}{2}\right)=6$ bivariate percentage plots.

### Permutation testing

Permutation testing between all pairwise comparisons for a user-selected summary statistic is performed by randomly shuffling the labels associated with each observed cell across all groups. Permutation testing is set to 500 permutations by default but can be adjusted by the user.

### Spectral karyotyping (SKY)

Copy number information is extracted from SKY data hosted within Microsoft Excel files in ISCN format using regular expressions.

### Single cell whole genome sequencing

Within aneuvis, copy number output in browser extensible data (BED) format is converted to a whole-chromosome summary copy number computed using a weighted average, where the inferred copy number at each bin along a chromosome contributes proportionally to the size of each bin (in base pairs). The weighted average is rounded to the nearest integer to obtain the chromosome copy number.

For the usage scenario, low-coverage single cell whole genome sequencing (sc-WGS) (0.01x) was generated from 27 young and 56 senescent IMR90 cells (for a total of 83 cells) across two sequencing runs. IMR90 cells were obtained from American Type Culture Collection (ATCC) (CCL-186). BAM files generated from the Torrent Suite software were converted to .bed files using the bedtools2 bamToBed function. Bed files were uploaded into Ginkgo’s user interface [16] with variable bin sizes of approximately 2.5 megabases (MB) and based on simulations of 150 bp reads with global segmentation [15]. The copy number matrix output from Ginkgo was used as input into aneuvis. Ginkgo copy number output and bed files are available at a Ginkgo-generated permalink [23].

### Experimental cell culture and four-color interphase FISH

Young and senescent IMR90 cells were generated and analyzed by four-color interphase FISH, as described previously [12]. Images representing nuclei were randomly acquired and saved as .tiff composite files for both young (*N* = 406) and senescent (*N* = 396) cells. Images were visually inspected and FISH signals manually counted blindly for both chromosomes 9 and 12 within a nucleus, as described previously [12].

### Example data

Example data using three treatment groups for each type of experimental input (FISH, SKY, and sc-WGS) are available through the aneuvis web application. Example FISH and SKY datasets represent ploidy counts that were manually generated to show varying degrees of severity across treatments. The example sc-WGS dataset is a breast cancer single cell dataset taken from Ginkgo [15, 24]. Artificial labels (Control, Treatment A, Treatment B) were added to all three example datasets to simulate treatments of varying severity.

### Summary of ginkgo output

Bed files from 83 cells were uploaded into ginkgo and processed as described in the “*Single cell whole genome sequencing*” section above. Screenshots were taken from each of the four sections of the Ginkgo output, described below. First, a “tree-display” within Ginkgo showcases a dendrogram of all cells based on genome-wide copy number status similarity (Fig. 6a, left side). Second, the “processed-data” section (Fig. 6a, right side) contains summarized copy number data in various formats that are available for download. The integer “copy number” state file from this section can be used as input into Aneuvis for further statistical analysis and visualization, particularly if different treatment groups were a part of the experimental design. Third, a series of heatmaps displays the copy number state or the number of reads from each cell at each bin in the genome (Fig. 6b). Fourth, a “summary” section shows a copy number scatterplot for each input .bed or .bam file alongside quality control summaries, such as the number of reads per file (Fig. 6c). Graphical outputs from selected files can also be generated from these copy number or quality control metrics. All visualizations are available in their original format at a Ginkgo-generated permalink [23].

## Additional file

Additional file 1: Aneuvis pdf output of graphics from the “Visualizations” tab. (PDF 95 kb)

## Abbreviations

ANCA: Average Number of Copy Number Alterations
BAM: Binary Alignment Map
BED: Browser Extensible Data
CGH: Comparative Genomic Hybridization
CNV: Copy Number Variation
DNA: Deoxyribonucleic acid
FISH: Fluorescence In Situ Hybridization
GUI: Graphical User Interface
QDV: Quantifying the Degree of numerical chromosomal Variation
sc-WGS: Single cell Whole Genome Sequencing
SKY: Spectral Karyotyping
W-CIN: Whole Chromosome Instability

## References

[1] Taylor AM, Shih J, Ha G, Gao GF, Zhang X, Berger AC (2018). Genomic and functional approaches to understanding cancer aneuploidy. Cancer Cell.

[2] Geigl JB, Obenauf AC, Schwarzbraun T, Speicher MR (2008). Defining ‘chromosomal instability. Trends Genet.

[3] Sansregret L, Vanhaesebroeck B, Swanton C (2018). Determinants and clinical implications of chromosomal instability in cancer. Nat Rev Clin Oncol.

[4] Bakker B, van den Bos H, Lansdorp PM, Foijer F (2015). How to count chromosomes in a cell: an overview of current and novel technologies. BioEssays.

[5] Bakker B, Taudt A, Belderbos ME, Porubsky D, Spierings DCJ, de Jong TV (2016). Single-cell sequencing reveals karyotype heterogeneity in murine and human malignancies. Genome Biol.

[6] Piqué D. Aneuvis: Web-based visualization of aneuploidy in single cells [internet]. Available from: https://dpique.shinyapps.io/aneuvis/. [Cited 26 Nov 2018]

[7] Bayani J, Paderova J, Murphy J, Rosen B, Zielenska M, Squire JA (2008). Distinct patterns of structural and numerical chromosomal instability characterize sporadic ovarian cancer. Neoplasia.

[8] Blegen H, Will JS, Ghadimi BM, Nash H-P, Zetterberg A, Auer G (2003). DNA amplifications and aneuploidy, high proliferative activity and impaired cell cycle control characterize breast carcinomas with poor prognosis. Anal Cell Pathol.

[9] Ried T, Heselmeyer-Haddad K, Blegen H, Schröck E, Auer G (1999). Genomic changes defining the genesis, progression, and malignancy potential in solid human tumors: a phenotype/genotype correlation. Genes Chromosom Cancer.

[10] Hamilton N (2018). Ggtern: an extension to “ggplot2”, for the creation of ternary diagrams. R Packag.

[11] Benjamini Y, Hochberg Y (1995). Controlling the false discovery rate: a practical and powerful approach to multiple testing. J R Stat Soc B.

[12] Andriani GA, Almeida VP, Faggioli F, Mauro M, Li Tsai W, Santambrogio L (2016). Whole chromosome instability induces senescence and promotes SASP. Sci Rep.

[13] Knierim E, Schwarz JM, Schuelke M, Seelow D (2013). CNVinspector: a web-based tool for the interactive evaluation of copy number variations in single patients and in cohorts. J Med Genet.

[14] Seelow D. CNVinspector [Internet]. Available from: http://www.cnvinspector.org/. [Cited 26 Nov 2018]

[15] Garvin T, Aboukhalil R, Kendall J, Baslan T, Atwal GS, Hicks J (2015). Interactive analysis and assessment of single-cell copy-number variations. Nat Methods.

[16] Garvin T, Aboukhalil R, Kendall J, Baslan T, Atwal GS, Hicks J, et al. Ginkgo: interactive analysis and assessment of single-cell copy-number variations [internet]. Available from: http://qb.cshl.edu/ginkgo/. [Cited 26 Nov 2018] 10.1038/nmeth.3578 PMC4775251 26344043

[17] Tebel K, Boldt V, Steininger A, Port M, Ebert G, Ullmann R (2017). GenomeCAT: a versatile tool for the analysis and integrative visualization of DNA copy number variants. BMC Bioinformatics.

[18] GenomeCAT - a versatile tool for the analysis and integrative visualization of DNA copy number variants [internet]. Available from: http://genomecat.github.io/genomeCATSuite/. [Cited 26 Nov 2018] 10.1186/s12859-016-1430-x PMC5217618 28061750

[19] van Riet J, Krol NMG, Atmodimedjo PN, Brosens E, van IJcken WFJ, Jansen MPHM (2018). SNPitty: An Intuitive Web Application for Interactive B-Allele Frequency and Copy Number Visualization of Next-Generation Sequencing Data. J Mol Diagn.

[20] SNPitty: An intuitive web-application for interactive B-allele frequency and copy-number visualization of next generation sequencing data [Internet]. Available from: https://bitbucket.org/ccbc/snpitty. [cited 26 Nov 2018] 10.1016/j.jmoldx.2017.11.011 29305224

[21] Allen EG, Freeman SB, Druschel C, Hobbs CA, O’Leary LA, Romitti PA (2009). Maternal age and risk for trisomy 21 assessed by the origin of chromosome nondisjunction: a report from the Atlanta and National Down syndrome Projects. Hum Genet.

[22] Recio R, Robbins WA, Borja-Aburto V, Morán-Martínez J, Froines JR, Hernández RM (2001). Organophosphorous pesticide exposure increases the frequency of sperm sex null aneuploidy. Environ Health Perspect.

[23] Ginkgo analysis of young and senescent fibroblasts [Internet]. Available from: http://qb.cshl.edu/ginkgo/?q=results/cutdaQTf5QubHWDcGxeX. [Cited 26 Nov 2018]

[24] Navin N, Kendall J, Troge J, Andrews P, Rodgers L, McIndoo J (2011). Tumour evolution inferred by single-cell sequencing. Nature.

[25] Piqué D. Aneuvis Github repository [internet]. Available from: https://github.com/dpique/aneuvis. [Cited 2018 Nov 26]

