# Supplementary material for: Aneuvis: web-based exploration of numerical chromosomal variation in single cells
Source: BMC Bioinformatics. 2019 Jun 17;20:336. doi: 10.1186/s12859-019-2842-1 (PMC6580570; doi:10.1186/s12859-019-2842-1)

Aneuvis Visualizations Report

aneuvis 0.7

November 26, 2018

This report was automatically generated from Aneuvis, a web tool for analyzing chromosomal number variation in single cells. Aneuvis summarizes chromosomal copy number data from 3 potential sources: FISH, SKY, and WGS.

Scores by Group

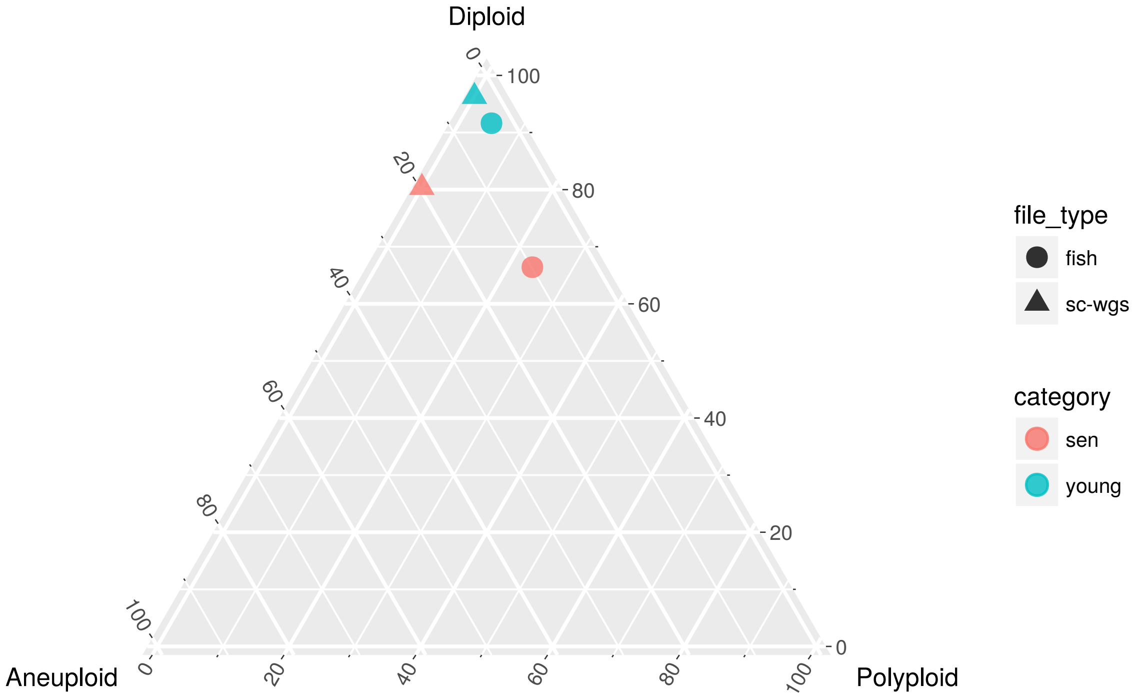

Aneuploidy vs. Heterogeneity Scatterplots

Scores by Group

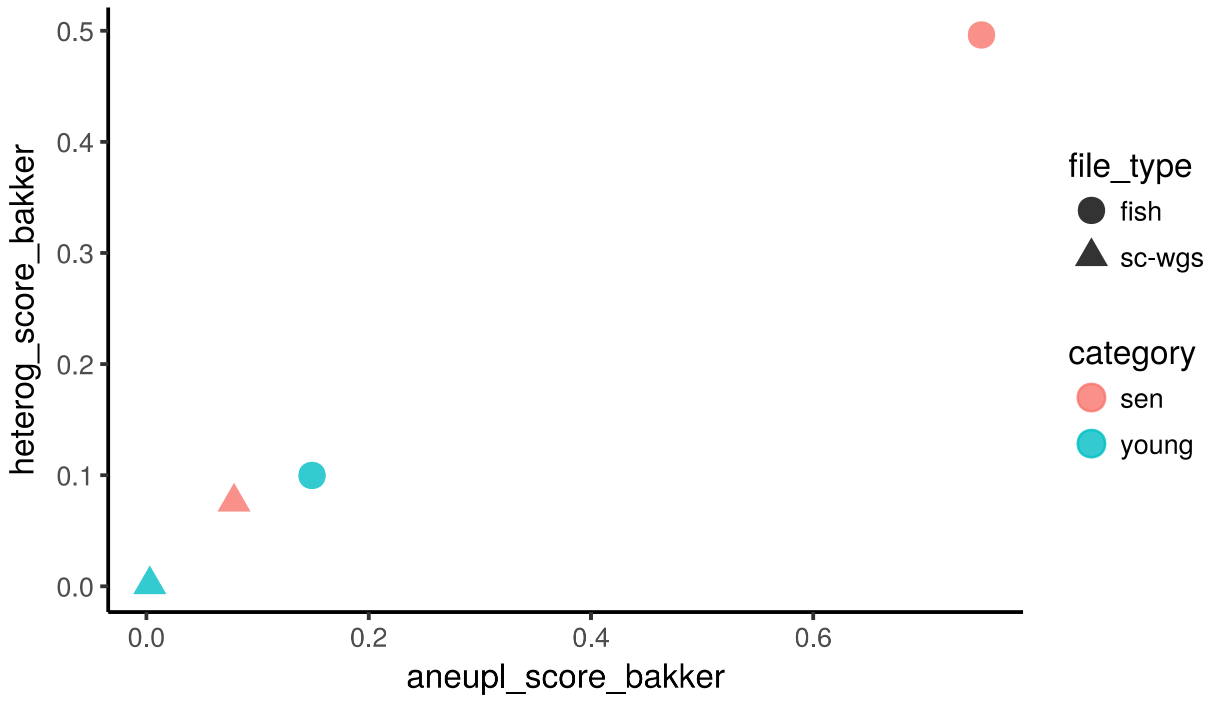

Scores by Group and Chromosome

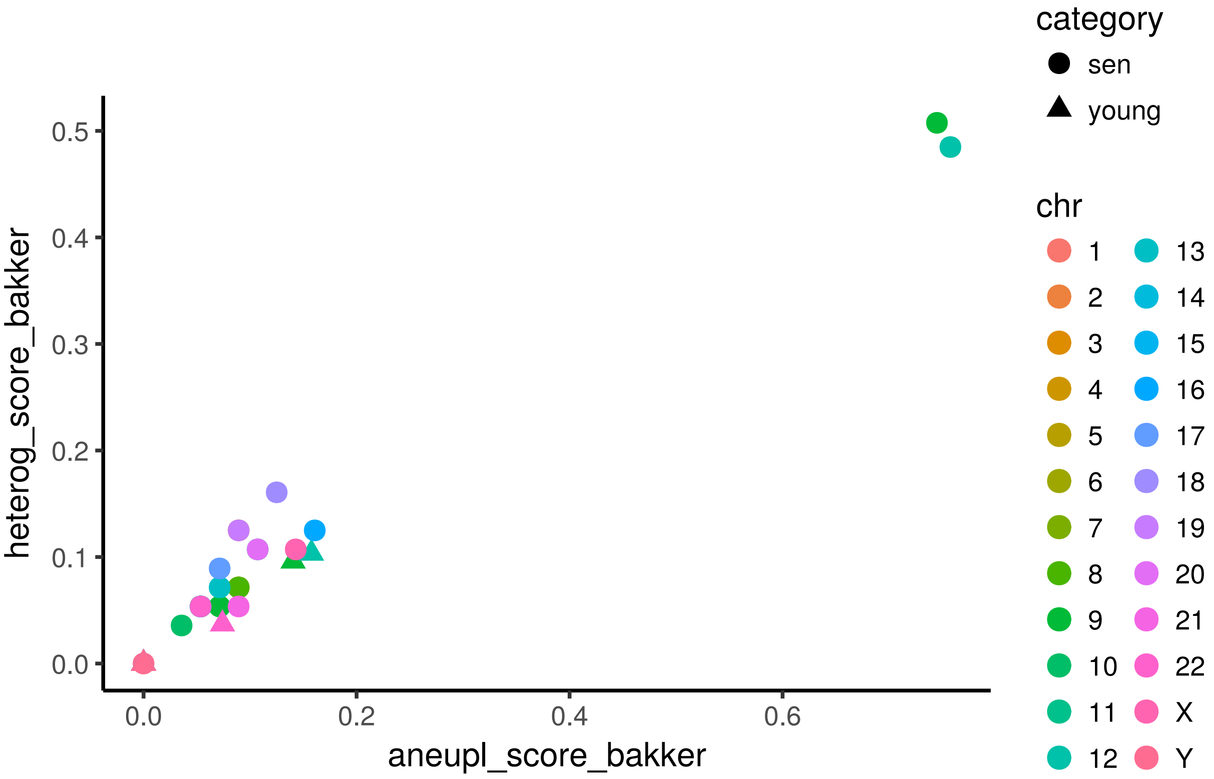

Datatype-specific plots

sc-WGS heatmap

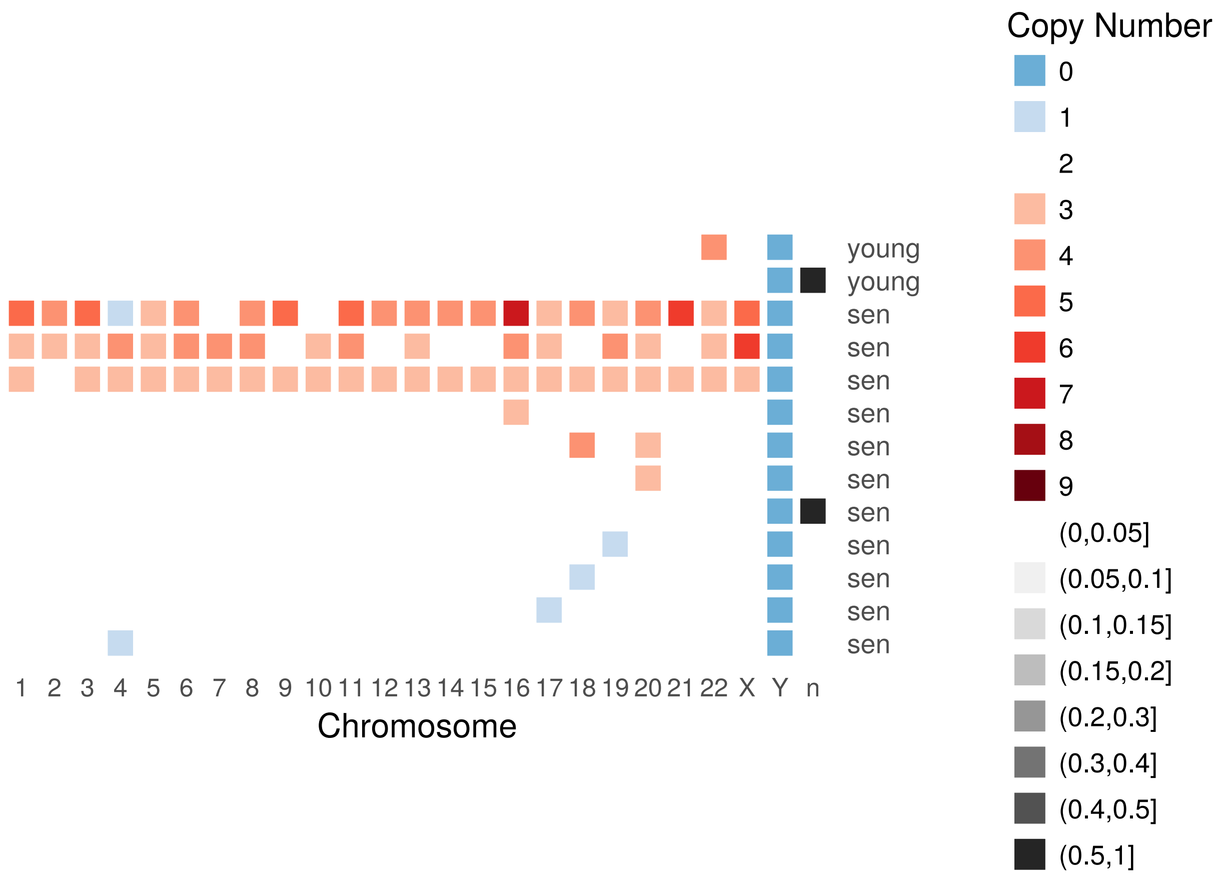

SKY heatmap

```
## [1] "no sky data uploaded"
```

FISH gridplots

% aneuploidy across 396 observations
file: sen

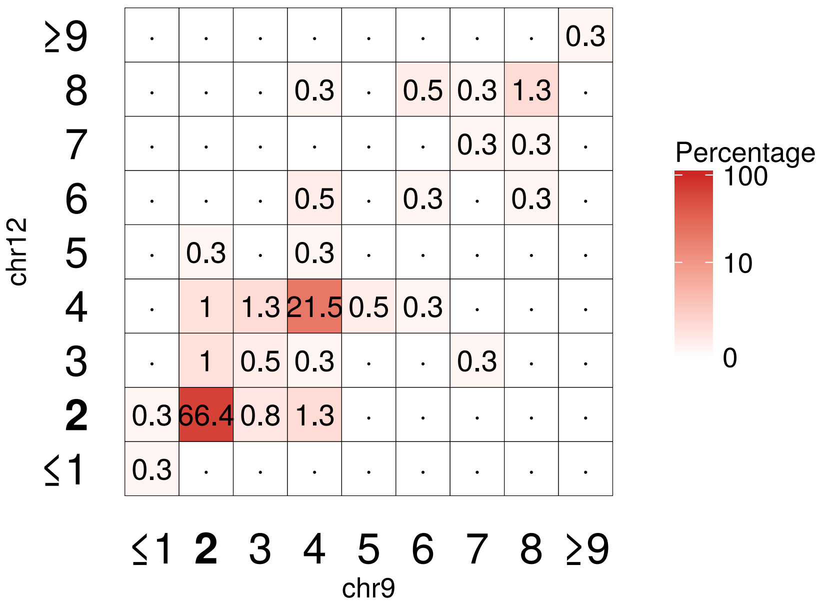

% aneuploidy across 406 observations
file: young

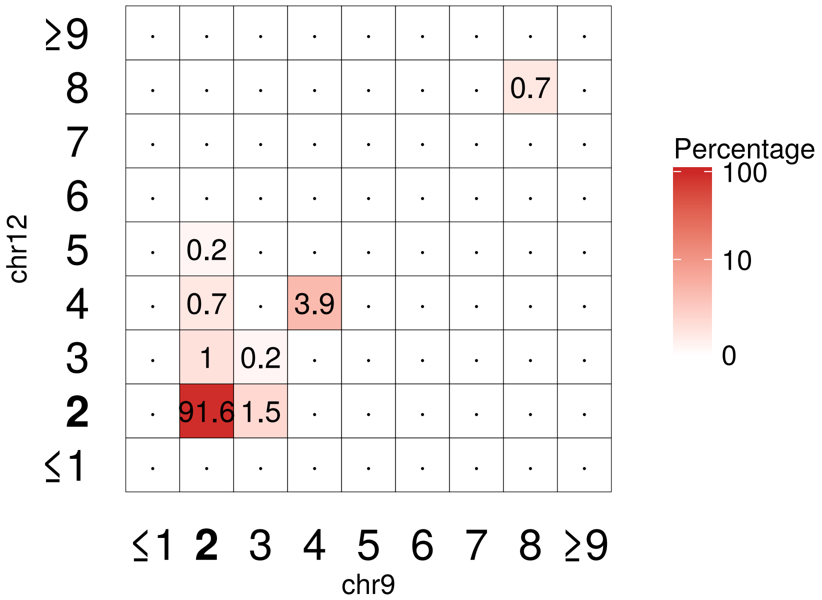

Supplement: Supplementary file 1 — Aneuvis pdf output of graphics from the “Visualizations” tab. (PDF 95 kb) [file 12859_2019_2842_MOESM1_ESM.pdf]
